# The relationship between schizophrenia and rheumatoid arthritis revisited: Genetic and epidemiological analyses

**DOI:** 10.1002/ajmg.b.32282

**Published:** 2015-02-05

**Authors:** Jack Euesden, Gerome Breen, Anne Farmer, Peter McGuffin, Cathryn M Lewis

**Affiliations:** ^1^ MRC SGDP Centre Institute of PsychiatryKing's College LondonLondonUK; ^2^ Department of Medical and Molecular GeneticsKing's College LondonLondonUK

## Abstract

Epidemiological studies are inconsistent on the relationship between schizophrenia (SCZ) and rheumatoid arthritis (RA). Several studies have shown that SCZ has a protective effect on RA, with RA occurring less frequently in SCZ cases than would be expected by chance, whilst other studies have failed to replicate this. We sought to test the hypothesis that this effect is due to a protective effect of SCZ risk alleles on RA onset. We first reviewed the literature on the comorbidity of RA and SCZ and performed a meta‐analysis. We then used polygenic risk scoring in an RA case control study in order to investigate the contribution of SCZ risk alleles to RA risk. Meta‐analysis across studies over the past half‐century showed that prevalence of RA in SCZ cases was significantly reduced (OR = 0.48, 95% CI: 0.34–0.67, p  < 0.0001). The relationship between SCZ genetic risk and RA status was weak. Polygenic risk of SCZ explained a small (0.1%) and non‐significant (p = 0.085) proportion of variance in RA case control status. This relationship was nominally positive, with RA cases carrying more SCZ risk alleles than controls. The current findings do not support the assertion that the relationship between RA and SCZ is explained by genetic factors, which appear to have little or no effect. The protective effect of SCZ on RA may be due to environmental factors, such as an anti‐inflammatory effect of anti‐psychotic medication or merely due to confounding limitations in study designs. © 2015 The Authors. *American Journal of Medical Genetics Part B: Neuropsychiatric Genetics* published by Wiley Periodicals, Inc.

**Abbreviations**
SNP: Single Nucleotide Polymorphism

## INTRODUCTION

Rheumatoid arthritis (OMIM 180300) and schizophrenia (OMIM 181500) are, superficially, remarkably different disorders. They have similar prevalences; rheumatoid arthritis (RA) has an estimated point prevalence 0.6% [Helmick et al., 2008], whilst schizophrenia (SCZ) has an estimated point prevalence of 0.46% [Saha et al., 2005]Saha et al., 2005). Lifetime prevalence for these disorders is substantially harder to measure, especially RA due to its later age at onset, however estimates for the lifetime prevalence of SCZ are as high as 0.72% [Saha et al., 2005]. Furthermore, both SCZ and RA show familial patterns of aggregation – heritability estimates for SCZ (0.81, 95% CI: 0.73–0.90) and RA (0.65, 95% CI: 0.50–0.77) are substantial [MacGregor et al., 2000][Sullivan et al., 2003]Sullivan et al., 2003). This implies a complex genetic aetiology, in which many risk alleles of small effect size can aggregate in individuals to modulate their risk of developing a disorder. Alongside its familial pattern of aggregation, schizophrenia also shows an unusual aggregation of comorbidities with many autoimmune disorders, such as Sjögren's Syndrome (OMIM %270150) [Eaton et al., 2006].

The relationship between SCZ and RA is much less clear, with many studies finding no evidence of a significant association (Eaton et al., 2006). Here we review the findings of such studies in order to evaluate the veracity of this relationship. RA seems to be protective for SCZ, with studies reporting an OR for RA status in schizophrenia patients as low as 0.44 (95% CI 0.24–0.81). This suggests a substantial protective effect of the disorder [Mors et al., 1999]Mors et al., 1999). This may be due to some risk factor for RA reducing schizophrenia risk, or vice versa. In order to understand this better, we apply a statistical genetics technique – polygenic risk scoring – to dissect the genetic relationship between the two disorders.

We are interested in explaining this relationship on three levels. On a genetic level, we are interested in the predetermined risk profiles carried by various individuals throughout their lifetimes; specifically the variance in disease status explainable by an individual's risk allele count. Secondly, we are interested in an epidemiological perspective – to explain the pattern of disease status and onset amongst a population, via a meta‐analysis of studies investigating this. Finally we are interested in an aetiological perspective – the interaction between pre‐existing risk and modulating factors that act to precipitate disease onset; we will examine aetiological and genetic data in order to make inferences on the aetiology of these two disorders.

RA is a joint disorder characterized by an elevation in levels of immune activity (e.g. increased T‐cell proliferation) accompanied by painful, swollen, and ultimately, eroded and fused joints. Converging evidence from pharmacology, serology and genetics suggests that RA is an autoimmune disease. Its relatively high prevalence has made RA amenable to high throughput genetic studies, leading to the identification of, to date, 101 risk loci [Okada et al., 2014], providing invaluable clues to its aetiology. The strongest association for RA is in the Human Leukocyte Antigen (HLA) region. The HLA genes are located in the MHC region, on the short arm of chromosome 6 [Shiina et al., 2006], and are involved in adaptive immune response.

Schizophrenia is a psychiatric disorder, characterized by auditory hallucinations, delusions and disorganized speech. Historically, theories of psychiatric aetiology have been rooted in a Cartesian dichotomy, with disorders of the ‘mind’ predicted to have limited physiological aetiology or phenomenology [Kendler, 2012]. This has led to a number of environmental aetiologies proposed for schizophrenia – for example an environment with a high level of expressed emotion [Bebbington & Kuipers, 1994]. Despite this, there have been a number of studies arguing for an immune component to the aetiology of schizophrenia – this began with McGuffin et al 1978, based on serological studies. More recently, genome‐wide association studies (GWAS) have identified genetic markers showing a significant association with schizophrenia. These genetic markers, Single Nucleotide Polymorphisms (SNPs) are studied across the genome in order to fine‐map regions associated with disease and subsequently predict disease risk in other cohorts. Most robust amongst these associations is a region in the HLA, which shows strong association in all studies (Ripke, 2011; S.[Ripke et al., 2013].

### Summary

We therefore sought to examine evidence for an epidemiological link between SCZ and RA by meta‐analysis of studies investigating RA amongst SCZ patients. Given the polygenic architecture of these two disorders, we also investigated whether the genetic predictors influencing SCZ risk had an atypical distribution amongst RA patients.

## RESULTS

### Meta‐Analysis

**Figure 1:**
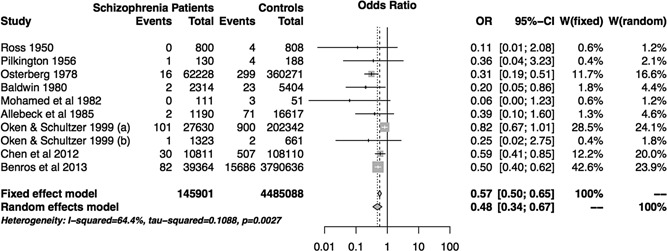
Meta‐analysis results. We identified 10 studies reported in 9 papers. Oken & Schultzer (a) compares schizophrenia vs other psychiatric patients in Canada meanwhile Oken & Schultzer (b) compares a similar sample in New York State. We present the RA prevalence (events) in SCZ cases vs controls across studies. W: weight for each study under random and fixed effects analysis.

After following a protocol specified below, we identified 10 studies reported in 9 papers reporting the prevalence of rheumatoid arthritis (RA) within a schizophrenia (SCZ) sample and a sample of controls. We used the results of these studies to perform a meta‐analysis (Fig. 1). Under a fixed effects model, SCZ status conferred an odds ratio of 0.57 (95% CI: 0.50–0.65, p  < 0.0001) on RA status, and an odds ratio of 0.48 (95% CI: 0.34–0.67, p  < 0.0001) under a random effects model, showing a significant protective effect of SCZ on RA status. There is statistically significant heterogeneity between studies (p = 0.0027) and therefore a random effects model is the most appropriate analysis approach.

These studies varied in their selection of controls – population controls, non‐schizophrenic psychiatric patient controls and non‐schizophrenic medical patients, and the most recent in a series of studies on a Danish population register comparing SCZ patients with population controls [Benros et al., 2013]. To maximise comparability, a major challenge in all epidemiological work, a number of studies use non‐schizophrenic psychiatric patients as controls. This allows the effect of schizophrenia to be studied in isolation. Five of these studies are based in individual psychiatric hospitals ‐ [Mohamed et al., 1982] Mohamed et al., 1982; [Ross et al., 1950]; [Pilkington, 1956]; [Oken & Schulzer, 1999] Ross et al., 1950). Two use record‐linkage methods [Baldwin, 1980]; [Osterberg, 1978] on international and Swedish populations respectively. Finally two studies used general medical disorder patients as controls [Allebeck et al., 1985]; [Chen et al., 2012].

All studies estimated nominally lower risks of RA in SCZ cases compared to controls, and this relationship was statistically significant in four studies [Baldwin, 1980]; [Chen et al., 2012]; [Benros et al., 2013]; [Osterberg, 1978], replicating the canonical ‘protective’ effect of SCZ on RA. It is notable that these four studies are the largest included, all using record linkage databases and thus the remaining 6 studies, which failed to find any significant effect, may have been simply under‐powered.

### Polygenic Risk Scoring

**Figure 2:**
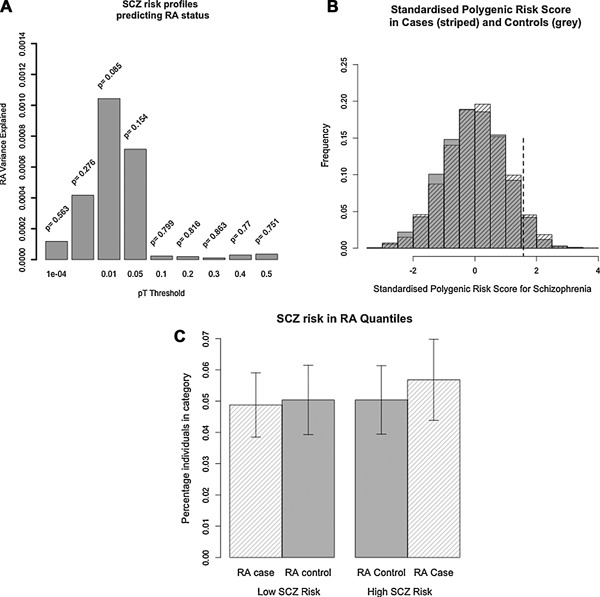
(a) Variance in RA status explained by SCZ polygenic risk scores in an independent test cohort. Scores are calculated across cutoff thresholds, pT. (b) Standardised polygenic risk score distribution at pT < 0.01 in RA cases (striped) and RA controls (grey). Dotted line ‐ top quantile (highest 5%) for SCZ risk amongst controls (standardised score > 1.57). 5.1% cases and 5.0% controls above this value. (c) SCZ risk in highest quantile (top 5%) and lowest quantile (bottom 5%) for SCZ risk between RA cases and controls.

We used published SCZ GWAS results (S. [Ripke et al., 2013] to calculate polygenic risk scores (PRS) in 1,989 RA cases and 1,588 controls. We used a series of thresholds, *p*_*T*_, to select SCZ risk alleles based on GWAS p‐value, and calculated risk scores for each of these risk allele sets (Table 1, Fig. 2a). SNPs associated with SCZ at *p*_*T*_ < 0.01 explain under 0.2% of the variance in RA status in the independent test cohort (Fig. 2b). This relationship is not statistically significant (p = 0.085) and is therefore no stronger than would be expected by chance. This is consistent with results using a considerably smaller SCZ sample (3,322 cases, 3,587 controls) as a discovery dataset [International Schizophrenia Consortium et al., 2009]. Standardised polygenic risk scores for SCZ at *p*_*T­*_ < 0.01 are approximately normally distributed, with no significant difference (p = 0.063) in mean score between cases (0.028) and controls (‐0.035), (Fig. 2c).

**Table 1:** Polygenic risk Scores for SCZ across thresholds and variance in RA status explained

Threshold, pT	Number of SNPs	Variance in RA status Explained, Pseudo R^2^	P‐Value
0.0001	82	0.0001	0.563
0.001	299	0.0004	0.276
0.01	1,393	0.0010	0.085
0.05	4,451	0.0007	0.154
0.1	7,396	0.0000	0.799
0.2	12,431	0.0000	0.816
0.3	16,708	0.0000	0.863
0.4	20,634	0.0000	0.770
0.5	24,122	0.0000	0.751

### Genetic Profile Risk Scoring

We calculated a measure of SCZ genetic risk in our RA cases and controls using the panel of SNPs proposed by Ayalew et al, identifying proxies where necessary using SNAP [Johnson et al., 2008];[Ayalew et al., 2012]. After QC, we obtained genotypes, imputed genotypes or proxies for 257 SNPs. SCZ genetic risk did not predict RA status – after adjusting for population structure, p = 0.858. We further explored the relationship between this panel of SNPs and RA, using SNP‐based and gene‐based summary statistic analyses (supplementary 8), and demonstrate that they do not show significant association with RA – SNP‐based p‐value = 0.13, gene‐based p‐value = 0.604

### Direction of Effect

We compared the direction of effect of risk alleles for SCZ and RA using published GWAS results for each (S.[Stahl et al., 2010];[Ripke et al., 2013]. After merging, 170,998 and 171,707 SNPs remained when clumping by RA p‐value and SCZ p‐value respectively, in order to obtain SNPs in approximate linkage equilibrium. The lack of association between SCZ alleles and RA alleles was confirmed through direction of effect analysis. We found no evidence for the proportion of alleles with a shared direction of effect between RA and SCZ deviating from our expectation under the null (Table 2), using a Sign Test.

**Table 2:** Direction of effect sharing for SNPs in approximate linkage equilibrium, between SCZ and RA GWAS's

Clumped By	Threshold, p <	N SNPs	P, Pearson's χ^2^	Proportion SNPs in same direction
RA	0.01	5,063	0.670	0.492
	0.1	35,795	0.569	0.503
	0.2	61,785	0.238	0.502
	0.3	83,174	0.241	0.502
	0.4	101,328	0.199	0.503
	0.5	117,664	0.152	0.503
SCZ	0.01	1,784	0.845	0.484
	0.1	16,503	0.966	0.496
	0.2	33,388	0.200	0.499
	0.3	50,159	0.217	0.498
	0.4	67,313	0.285	0.498
	0.5	84,613	0.179	0.498

## DISCUSSION

Our SCZ polygenic risk scores analysis has shown that variance in RA status cannot be predicted or explained by burden of SCZ risk alleles genome‐wide. This is supported by an analysis of Genetic‐Profile Risk Scores. On considering the epidemiology of these two disorders, this finding is consistent with the notion that there is no ‘protective’ effect of SCZ on RA – one would not be predicted from genetic data alone. Despite this, we have also demonstrated through meta‐analysis that the negative association between the two disorders appears consistent across studies. Below we review the epidemiological and genetic evidence presented above, and propose some aetiological theories to reconcile them.

### Epidemiology

The protective effect of SCZ on RA is supported in our meta‐analysis, with an infrequency of RA in SCZ cases, which would not be predicted by chance. The possible protective effect of institutionalisation on RA status can be parsed from the effect of SCZ by looking at studies using institutionalised controls. Rothermich & Philips studied a prison population in order to investigate the protective effect of long term institutionalisation; although they found no significant relationship between RA and SCZ when using RA in prisoners as a control population, they found nominal, but non‐significant, evidence of a protective effect of SCZ on RA onset [Rothermich & Philips, 1963]. This is consistent with equally underpowered studies using general psychiatric samples as controls.

### Genetics

Both SCZ and RA have been associated with a number of risk alleles at genome‐wide significance. Converging evidence for a lack of shared genetic substrate between RA and SCZ comes from family studies of the two disorders [Benros et al., 2013]. The authors explored family history of SCZ (a proxy for SCZ risk allele burden) as a predictor of RA, finding no evidence of a significant association – the relative risk for family history of SCZ on RA risk was 0.94 (95% CI: 0.84–1.06).

### Aetiology

The aetiologies of both rheumatoid arthritis and schizophrenia are still topics of active research. Evidence for an autoimmune substrate to schizophrenia has coalesced in recent years, driven by the genome‐wide significant loci in the Major Histocompatibility Complex [Ripke., 2011; S.[Ripke et al., 2013]. This complements work in serological analysis of SCZ patients, notably [McGuffin et al., 1978], who found an increase in HLA‐BW5 and a decrease in HLA‐AW29 and HLA‐BW17 in serum of SCZ patients (McGuffin, Farmer, & Rajah, 1978).

The association between SCZ and immune‐related biomarkers may be reconciled as autoimmune over‐activity specific to a component of the nervous system. A systematic review of blood protein expression in SCZ patients found evidence of increased autoantibodies for the N‐methyl‐D‐Aspartate receptor (NMDA‐R) [Ezeoke et al., 2013]Ezeoke et al., 2013), which underlies the formation of associative memory by mediating the strengthening of synapses [Bannerman et al., 1995]Bannerman et al., 1995). An autoimmune pathology could therefore underlie damage to neural tissue, and therefore networks, resulting in the cognitive symptoms observed in schizophrenia [van den Heuvel et al., 2013].

Despite the plausibility of this model, we do not find evidence for a genetic overlap between SCZ and RA. This suggests that, if some of the biological pathways involved in RA and SCZ are shared, it may be environmental rather than genetic aberrations perturbing these. Negative results must always be viewed cautiously in the context of power, and we discuss this limitation below.

### Effect of Medication

We considered the epidemiological data on SCZ and RA in the light of their respective ages at onset. SCZ has a mean age at onset of around 26 years (95% CI 14.34 – 38.14) [Sham et al., 1994]. By contrast, RA has a much later age at onset, with the peak age at onset between 65–75 in men and 55–64 in women [Symmons and Deborah, 2002]. We considered that, by age at onset for RA, SCZ patients were likely to be medicated. If these two disorders do share an aetiological basis, antipsychotic medication may have a prophylactic effect on RA onset later in life.

The epidemiological studies presented above, exploring the relationship between SCZ and RA, do not stratify patients by medication status. It is unlikely, however, that medication status mediates the negative association. Chlorpromazine was first introduced clinically in the early 1950's, and clinical uptake of antipsychotics in the USA was gradual from the mid‐1950's to the mid‐1970 s [Shen, 1999]. Despite this, there is substantial evidence that typical antipsychotics such as haloperidol may have an anti‐inflammatory role that may protect against RA. Synovitis and CRP levels in RA patients has been observed to improve following administration of haloperidol for acute mania in case studies, and in blood cultures stimulated acute inflammation led to a marked inhibition of the release of TNF α and IL1‐β [Moots et al., 1999]. These inflammatory cytokines have been directly linked to RA [Elliott et al., 1995]Elliott et al., 1995;[McNiff et al., 1995]McNiff et al., 1995). Thus schizophrenia patients taking haloperidol may be protected from RA onset by the suppression of TNF‐α and IL1‐β levels.

### Limitations

We identify four main limitations in our study. Firstly, as presented above, SCZ and RA have substantially different ages at onset, and the former is associated with substantially reduced life expectancy [Crump et al., 2013]Crump et al., 2013); thus many SCZ patients may die before age at onset for RA. Many epidemiological studies above are unable to adjust for age amongst SCZ patients – in a record linkage paradigm, this data is not collected – and therefore we present unadjusted odds ratios for all studies. A ‘harvesting effect’ may confound the negative association between RA and SCZ [Sawchuk et al., 2013]Sawchuk et al., 2013); this is unlikely to account for the entire effect, as individual population registry studies, which collect sufficient data with sufficient power, replicate the negative association after adjusting for age [Benros et al., 2013]. Furthermore, work on the Swedish Population Register has replicated the protective effect of SCZ on subsequent RA diagnosis using Cox regression models and adjusting for age (Hazard Ratio = 0.69, 95% CI = 0.59–0.80) [Sellgren et al., 2014]Sellgren et al., 2014).

Our RA cases and controls present a second limitation. They are genotyped on different microarrays, so we can only use SNPs shared across both platforms for calculating SCZ polygenic risk scores. As polygenic risk scoring requires SNPs in approximate linkage equilibrium, the number of SNPs remaining in our test dataset for polygenic scoring is similar to what would be expected when using a sample genotyped on a single platform. Although our controls have been screened for Major Depressive Disorder (MDD, OMIM 608516) and are not a true population cohort, GWAS have been consistently shown to be underpowered to detect risk variants associated with MDD [MDD Working Group of the Psychiatric Genomics Consortium et al., 2013] and so this is unlikely to affect our results.

Power considerations are a persistent concern in polygenic risk scoring. Calculation of power requires a series of assumptions to be made on the underlying architecture of the diseases studied, such as the correlation between genetic effects in the discovery and test datasets. Power calculations (Supplementary 6) show it is likely that we would have sufficient power to detect an epidemiologically meaningful correlation in genetic effects – assuming genetic effects at 1% of SNPs, we have 80% power to detect a modest genetic effect correlation (magnitude = 0.078) at α = 0.05. Nevertheless, the possibility that our polygenic scoring results are a false negative is an important caveat.

Finally, as discussed above, RA and SCZ risk are both modulated by genotype at HLA loci. We have modelled this influence to an extent by including the most strongly associated SCZ risk SNP in this region in the calculation of polygenic risk scores. We estimated that SCZ status is protective for RA status with OR = 0.48 (95% CI: 0.34–0.67, p  < 0.0001). The effect sizes of risk alleles in complex disease genetics are substantially smaller than this ‐ the most significant MHC association with SCZ has an OR of 1.21 (S.[Ripke et al., 2013]. Therefore it is unlikely that SCZ risk at the MHC alone could mediate the epidemiological effect calculated in meta‐analysis above

### Summary

Despite the mounting evidence for an autoimmune aetiology in schizophrenia, and epidemiological literature on the co‐occurrence of these two disorders, we found no evidence for a shared genetic substrate between rheumatoid arthritis and schizophrenia, although this could be due to lack of power in the current samples. Epidemiological data may be confounded due to some protective effect acting to prevent onset of RA in high‐risk individuals. © 2015 Wiley Periodicals, Inc.

## MATERIALS AND METHODS

### Meta‐analysis

We performed a systematic review and meta‐analysis of studies investigating the prevalence of RA within SCZ patients. This was performed by searching Embase and Medline for articles published between 1945 and November 2013 containing the terms schiz$ AND rheuma$. We included only studies collecting data on RA within SCZ cases and a sample of SCZ controls. We restricted this to studies using population samples, non‐schizophrenic psychiatric patients or other physical disorder patients.

We included all Journal Articles and retained Reviews meeting these criteria. We then read the bibliographies of all reviews and included any articles with relevant abstracts. Finally we read all articles extracted and retained those containing epidemiological studies of RA and SCZ prevalences, which also reported RA prevalences for SCZ controls. We extracted the following data; study name, authorship and year, case and control sample size, RA incidence in each of these populations and selection criteria for controls.

We excluded case studies and studies that did not also collect controls (see S3 for full details of method used). Literature search, data extraction and quality assessment was performed in an unblinded manner by J.E. We combined studies and calculated meta‐analysis odds ratios under random effects and fixed effects using the R package meta.

### Genetic Data Used

As a SCZ discovery data set, we used the most recent publically available results of GWAS of schizophrenia from a meta‐analysis of the PGC1‐SCZ study and a Swedish cohort (S.[Ripke et al., 2013], (full details of cohort in S5). This reported the p‐value, odds ratio and test statistics for 9,898,079 SNPs imputed to the 1000 Genomes project [Siva, 2008]. For the RA target study, we used RA cases from the WTCCC study and controls from the RADIANT depression study. These controls were not included in the discovery study and therefore our discovery and test datasets are independent, as required for polygenic risk scoring. This contained data on 1,999 cases and 1,588 controls.

The WTCCC RA cases (n = 1,999) were collected across multiple UK studies co‐ordinated by the Arthritis Research Campaign's Epidemiology Unit [Wellcome Trust Case Control Consortium., 2007]. All cases satisfied the criteria for RA specified by the American College of Rheumatology [Arnett et al., 1988].

The 1588 RADIANT controls were collected from the staff and student body of King's College London or recruited via the Medical Research Council's general practice research framework [Lewis et al., 2010]. They were screened negative for a lifetime history of any psychiatric diagnosis, using a modified version of the Past History Schedule (P. McGuffin, Katz, & Aldrich, 1986) and all reported to be of white European ancestry.

### Cleaning Test Dataset

The RADIANT and WTCCC samples were genotyped on separate platforms (Illumina 610 quad bead and Affymetrix 500 k respectively), leading to a degree of attrition when merging datasets; after merging, 70,130 SNPs remained. We performed detailed quality control on the merged RADIANT‐WTCCC dataset. The final data set contained 1,989 RA cases and 1,588 controls (table S1) with genotype data on 69,621 SNPs.

### Cleaning Discovery Dataset

In the PGC and Swedish combined schizophrenia GWAS results as a discovery dataset, we removed SNPs with an info score less than 0.7, indicating poor imputation quality and SNPs not present in the cleaned test dataset. Finally, in order to obtain SNPs in approximate linkage equilibrium, the HLA region (26–33 Mb on chromosome 6) was omitted, except for most significant SNP in this region (rs2517611). We used p‐value‐informed clumping, extracting SNPs based on linkage disequilibrium (LD) in HapMap2 CEU samples. This left 24,126 independent SNPs in our discovery data set.

### Polygenic Risk Scoring

Polygenic risk scoring was performed in the RA test data set, based on SNPs extracted from the SCZ discovery data set meeting p‐value thresholds *p*_*T*_ of 0.001, 0.01, 0.05, 0.1, 0.2, 0.3, 0.4 and 0.5. At each threshold, *p*_*T*_, SNPs with SCZ association p‐values below the threshold were used to construct polygenic risk scores (PRS) for each individual in the RA test data set by summing the number of risk alleles at each SNP weighted by the natural logarithm of its odds ratio.

We then tested whether the SCZ PRS predicted variance in RA disease state in a logistic model, regressing disease state on PRS plus five ancestry‐informative dimensions accounting for population structure. The variance in disease state explained by this model was calculated as Nagelkerke's pseudo R^2^ (NR^2^). We report the difference in NR^2^ between this model and a model based on the ancestry‐informative dimensions alone.

### Genetic Profile Risk Scoring

We used the panel of 542 SNPs reported by Ayalew et al, which have been previously shown to serve as reliable predictors of SCZ status within independent cohorts and cohorts of different ethnicity [Ayalew et al., 2012], in order to construct genetic profile risk scores. We imputed our cases and controls to 1000Genomes and performed stringent QC. Of the 542 SNPs listed by Ayalew et al, we obtained genotypes or proxies with R^2^ > 0.6 for 257. We calculated weighted scores for SCZ genetic risk in our RA cases and controls using these SNPs and the effect sizes reported by Ayalew et al, and fitted a logistic regression model adjusting for population structure using 5 ancestry informative dimensions calculated on genotyped SNPs.

### Direction of Effect

In order to assess for consistency of direction of effect for SNPs between two schizophrenia and rheumatoid arthritis, we used published GWAS data from each disorder (S.[Stahl et al., 2010];[Ripke et al., 2013]. We performed quality control for imputation quality (as outlined above) and used p‐value informed LD clumping to obtain relatively independent SNPs, using the same protocol above. For each clumped GWAS, we merged with GWAS results for the other disorder, extracted all SNPs below a particular p‐value threshold, and classified SNPs as having the same direction of effect (both ORs > 1 for the same SNP allele), or different direction of effect. Consistency of SNP effect was tested for using Pearson's χ^2^ statistics, commonly termed a ‘sign test’.

## Supporting information

Additional supporting information may be found in the online version of this article at the publisher's web‐site.

Supporting Information.
