# Supplementary material for: The relationship between schizophrenia and rheumatoid arthritis revisited: Genetic and epidemiological analyses
Source: Am J Med Genet B Neuropsychiatr Genet. 2015 Feb 5;168(2):81–8. doi: 10.1002/ajmg.b.32282 (PMC4833173; doi:10.1002/ajmg.b.32282)
Supplement: Supplementary file 1 — Supporting Information. [file AJMG-168-81-s001.docx]

**Supplementary Materials:**

**Supplementary 1: Cleaning Test dataset**

Firstly, in order to account for any possible issues arising from using data from two different platforms, we analysed the WTCCC control dataset, genotyped on the Affymetrix 500k (3004 individuals, genotyped by the WTCCC, drawn from UK blood donors and the 1958 birth cohort, (Wellcome Trust Case Control Consortium, 2007)). We merged this dataset with the RADIANT controls, tested for association between the two data sets and removed any SNPs with p < 10^-5^ for association from subsequent analyses. We also removed any SNPs with different physical positions between the two platforms, any SNPs with MAF < 0.05 in either control group, any SNPs with a difference in frequency > 0.15 between the two groups and any SNPs failing our general QC criteria outlined below. We removed any SNPs with genotyping rate < 0.99 or a P-value for Hardy-Weinberg Equilibrium p < 5.7x10^-7^. We also removed individuals with missingness > 0.03. This left 69,623 SNPs. Finally, we removed 10 SNPs reaching genome-wide significance (p < 5.7 x 10^-8^ which were not within 1 Mb of previously reported genome-wide significant loci for rheumatoid arthritis (Eyre et al., 2012), leaving 69,613 SNPs. We tested for cryptic relatedness, however no pairs of individuals met our exclusion criteria ($\hat{\pi}$ > 0.2). The genomic control λ value between controls on the two chips to 1.097 indicating good consistency (**fig S1.a**).

The primary criticism of our approach, using two different chips, would be the concern that between-chip differences, unaccounted for by covariates for ancestry, would lead to spurious results. We tested this by investigating the genomic inflation, λ, after using 20 dimensions accounting for population structure (calculated using MDS) to covary for effects of ancestry. We calculated dimensions in PLINK by first producing a set of genome-wide SNPs in linkage equilibrium. We removed the MHC (an area of high linkage disequilibrium) and pruned using a sliding window of 50bp, moving by 5bp, and removing SNPs which produce a variance inflation factor (VIF) greater than 2 within that window. This left 47,951 SNPs, which were used to calculate dimensions.

We calculated 20 dimensions, and then regressed control group membership (RADIANT vs WTCCC1) on genotype plus increasing number of dimensions, noting genomic inflation for each model. The genomic inflation factor, a measure of population structure, calculated using the median chi-squared statistic, was λ = 1.0395 when using 5 dimensions as covariates, indicating that there was minimal population structure which couldn’t be accounted for by the use of dimensions accounting for population structure. This is a critical justification for our rationale, as we rely on our ability to assume that differences between cases and controls in our test dataset are due to alleles differentially associated with RA, rather than simply physical differences genotyping chips used.

This case-control analysis is also used to determine the number of eigenvectors necessary to account for population stratification between our case and control datasets. We used multi-dimensional scaling (MDS) to calculate eigenvectors for our merged dataset. MDS requires SNPs in linkage equilibrium. We therefore removed the MHC (26 – 33 mb on chromosome 6, an area of high linkage disequilibrium), and pruned the remaining SNPs using PLINK-1.07 under the protocol outlined above - using a sliding window of 50bp, moving by 5bp, and removing SNPs which produce a variance inflation factor (VIF) greater than 2 within that window.

| **S1.a** | **b** |
| --- | --- |
| 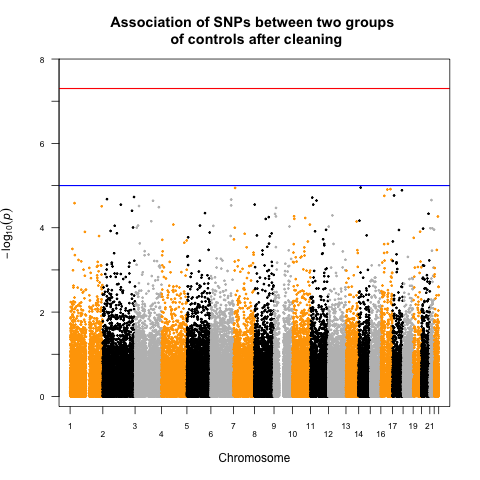 | 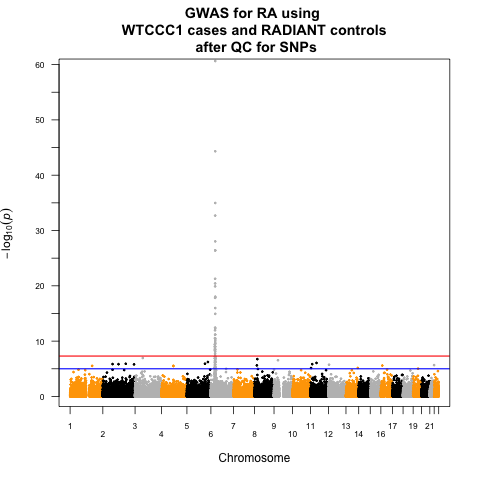 |
| **c** |  |
| 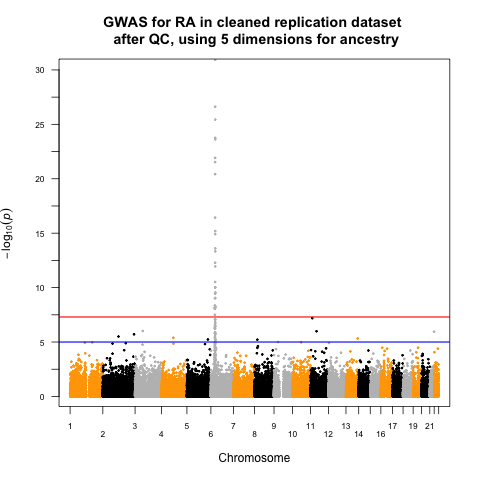 |  |
| **Fig S1**:  **a.** Genome-wide association between the controls used in the test dataset (from the RADIANT study) and the WTCCC controls. We were interested in removing SNPs differing substantially between the platforms these two groups were genotyped on. After cleaning, there was no substantial difference between the two groups  **b.** GWAS of RADIANT controls vs WTCCC-RA cases. We have replicated the WTCCC’s original (2007) result, with a substantially associated region in the MHC on chromosome 6  **c.** Manhattan Plot of association with RA for with SNPs in cleaned test dataset. P-values from logistic regression after using 5 dimensions to account for population structure | |

We calculated the first 20 dimensions that mapped identity by similarity across independent SNPs. The first two dimensions showed substantial deviation from expectation (**fig S2**), and so we removed individuals with a score on dimension 1 less than -0.06, or on dimension 3 less than -0.06. This produced a more conventional plot of all the first 4 dimensions. We therefore had 1,989 cases and 1,588 controls remaining – this left 3,577 individuals in our test dataset.

**Fig S2:** Axes of dimensions accounting for population structure; in all graphs, cases are in red, controls are in black, Europeans (CEU) are in green, Chinese (CHB) are dark blue, Japanese (JPT) are light blue and Yorubans (YRI) are purple. The complete RADIANT controls, Wellcome Trust Cases and HapMap2.3 samples are presented on the left column. We removed individuals with a score on dimension 1 less than -0.06, or on dimension 3 less than -0.06.The remaining individuals are presented on the right column.

This is broadly in line with the Principal Component plots that should be expected under a null assumption of no population stratification. We then ran a series of logistic regression models in PLINK adding increasing numbers of dimensions used as covariates. When using 5 dimensions as covariates, genomic inflation was λ = 1.095, indicating good control of ancestry or platform differences in the merged data sets. The results of this association test are presented as a Manhattan plot (**fig S1.c)**. The individuals used in the test dataset are summarised below **(table S1)**.

| RA Cases | | | | RA Controls | | | | Total |
| --- | --- | --- | --- | --- | --- | --- | --- | --- |
| **Male** | Female | Unknown | Total | Male | Female | Unknown | Total |  |
| **498** | 1491 | 0 | 1989 | 595 | 993 | 0 | 1588 | 3577 |

**Table S1:** Distribution of sex and affection status in test dataset

**Supplementary 2: Polygenic Risk Scoring excluding MHC SNP**

We repeated genome-wide polygenic scoring without the addition of any MHC-SNP. The results of this are below (**table S2, fig S3.a).**

| Threshold, *p_T_* | Number of SNPs included | Variance in RA status explained, Pseudo R^2^ | P-Value |
| --- | --- | --- | --- |
| 0.0001 | 81 | 0.0001 | 0.519 |
| 0.001 | 298 | 0.0004 | 0.259 |
| 0.01 | 1,392 | 0.0011 | 0.072 |
| 0.05 | 4,450 | 0.0007 | 0.152 |
| 0.1 | 7,395 | 0.0000 | 0.858 |
| 0.2 | 12,430 | 0.0000 | 0.938 |
| 0.3 | 16,707 | 0.0000 | 0.944 |
| 0.4 | 20,633 | 0.0000 | 0.826 |
| 0.5 | 24,121 | 0.0000 | 0.800 |

**Table S2**: Proportion of variance in RA status explained by polygenic risk score for SCZ calculated at different thresholds, *p^­^_T_*_,_ using SNPs genome-wide excluding the MHC-region

The upper 5% tails of the distribution, which capture those with high SCZ PRS scores, had similar proportions of RA cases and controls (cases: 5.7%; 95% CI 4.6% - 6.7% compared to controls 5.0%, 95% CI 4.0% - 6.1%) and similar results were seen in the lower 5% tail (**Figure S3.b**). These results suggest that genetic factors do not predict any epidemiological patterns of comorbidity between RA and SCZ.

| **S3.a** | **b** |
| --- | --- |
|  |  |
| **Fig S3.**  **a.** Variance in RA status explained by polygenic risk scores for SCZ calculated using different cut-off thresholds.  **b.** Proportion of RA cases (lined) and RA controls (grey) in lowest SCZ risk quantile (standardized PRS < -1.66) and in highest SCZ risk quantile (standardized PRS > 1.58). | |

We explored the logistic model regressing RA status on 5 dimensions accounting for population structure and standardised polygenic risk score (p_T_ < 0.01) for schizophrenia. In this model, the beta for PRS was 0.06. That is to say, a two standard deviation increase in polygenic risk for SCZ has an odds ratio of 1.03 (95% CI: 0.997 – 1.068) for RA risk.

**Supplementary 3: Systematic Review and Meta-analysis**

We performed a single meta-analysis of all studies extracted above. In addition to analysing the full systematic review results, we subdivided studies by control population. To account for effects of long term institutionalisation and aid in sample collection, many authors used non-schizophrenic psychiatric patients as their control group – a meta-analysis of the incidence of RA between SCZ patients and these reference patients *only* is shown below **(fig S4)**. The effect of SCZ on reducing RA incidence is preserved, indicating a main effect of SCZ, rather that psychiatric illness in general, driving this association.

The negative association between SCZ status and RA prevalence remained statistically significant, random effects OR = 343 (95% CI: 0.163 – 0.720, p = 0.0047). There was significant heterogeneity between studies (p = 0.0019).

|  |
| --- |
| **Fig S4**: RA prevalence (events) in SCZ cases and non-SCZ psychiatric patients. The original significant protective effect of SCZ on RA reported above was replicated here |

**Supplementary 4: Using PGC1 SCZ only as Discovery Dataset**

**4.1 Cleaning Discovery Dataset**

In order to use the PGC schizophrenia GWAS results **(table S3)** as a discovery dataset, a few quality control procedures are necessary. We removed SNPs from the GWA results with an info score less than 0.7, indicating poor imputation quality. We then removed SNPs not present in the cleaned test dataset. Finally, in order to obtain SNPs in approximate linkage equilibrium, we used p-value and LD-informed clumping, extracting SNPs based on LD in HapMap2 CEU samples. Specifically, we selected the SNP with the lowest p-value in each LD block of r^2^ < 0.1, length ≤250kb, leaving 23,150 independent SNPs in our discovery data sets.

| SCZ Cases | | | | SCZ Controls | | | | Total |
| --- | --- | --- | --- | --- | --- | --- | --- | --- |
| Male | Female | Unknown | Total | Male | Female | Unknown | Total |  |
| 4,731 | 3,106 | 22 | 8,442 | 10,449 | 10,933 | 15 | 21,397 | 29,833 |

**Table S3:** Sample characteristics of PGC1 SCZ Study, used as discovery sample for PRS in this section

**4.2 Polygenic Scoring**

After all quality control, our final test dataset contained genotype data on 1989 cases and 1588 controls. After removing the MHC region (26-33mb on chromosome 6), 23,301 SNPs remained. We calculated the proportion of variance in RA status explained by these polygenic risk scores after removal of the MHC (**table S4, fig S5a**)

We standardised polygenic risk score (*p_T_* < 0.05) for schizophrenia – the most predictive threshold (**fig S5.b).**  Standardised polygenic scores for SCZ risk were significantly higher in RA cases than controls (p = 0.0127).

| **S5.a** |
| --- |
|  |
| **b** |
|  |
| **Figure S5**:  **a.** Variance in RA status explained by polygenic risk scores for SCZ calculated using different cut-off thresholds.  **b**. Distribution of standardized polygenic risk scores in cases (light grey) and controls (dark grey). |

| Threshold P-value, *p_T_* | Number of SNPs | Additional variance explained | P-value of Polygenic Risk Score |
| --- | --- | --- | --- |
| 0.001 | 276 | 0.000275 | 0.568 |
| 0.01 | 1,274 | 0.000849 | 0.376 |
| 0.05 | 4,059 | 0.00204 | 0.120 |
| 0.1 | 6,880 | 0.00166 | 0.0160 |
| 0.2 | 11,778 | 0.000929 | 0.0300 |
| 0.3 | 15,955 | 0.00143 | 0.0440 |
| 0.4 | 19,812 | 0.00154 | 0.0363 |
| 0.5 | 23,301 | 0.00165 | 0.0302 |

**Table S4**: Polygenic Scoring testing variance in RA status explained by SCZ polygenic risk scores, using PGC1 SCZ data only as a discovery dataset

**Supplementary 5: PGC1 + Swedish Data – Sample Characteristics**

The PGC1+Swedish dataset contains summary GWAS results for a meta-analysis of two GWAS studies of schizophrenia. The first is the PGC1 SCZ results reported above (table S3) excluding the samples from Sweden – 8,832 cases, 12,067 controls. Secondly the authors performed GWAS of 5,001 cases and 6,243 controls. The results of this analysis are publically available via the PGC. <https://pgc.unc.edu/Sharing.php> and are analysed here as available on Feb 9^th^ 2014.

| **Swedish Sample Characteristics** | **Cases** | **Controls** |
| --- | --- | --- |
| Proportion Male | 0.595 | 0.512 |
| Median Age | 54 (45 – 62) | 57 (48-65) |

**Table S5:** Swedish sample characteristics. Meta-analysed alongside PGC1 results in order to produce Swedish+ PGC1 dataset.

**Supplementary 6: Power Calculations for PRS**

We used the polygenescore software developed by Dudbridge to calculate power in our polygene scoring analysis of SCZ risk in RA cases (Dudbridge, 2013). We calculated power to detect shared risk alleles between RA and SCZ at our most predictive polygene score threshold (pT < 0.01). This calculation takes the following parameters (**table S6**).

We tested three values of potential correlations between genetic effect sizes, based on genetic pleiotropy work by Lee et al (Lee, Yang, Goddard, Visscher, & Wray, 2012). We calculated power at α = 0.05 for genetic correlations of 0.05, 0.1 and 0.15

| Parameter | Value |
| --- | --- |
| Number of samples in discovery dataset | 13,833 cases,  18,310 controls |
| Number of SNPs in analysis | 1,393 |
| Number of samples in test dataset | 1,989 cases,  1,588 controls |
| Correlation between genetic effects in discovery dataset (SCZ GWAS) and test dataset (RA GWAS) | 0.05, 0.10, 0.15 |
| Variance in Discovery dataset explained by all genetic effects (GREML estimate from PGC 2013) | 0.26 |
| Variance in Test dataset explained by all genetic effects (GREML estimate from Stahl et al 2012) | 0.18 |
| Prevalence of SCZ | 0.01 |
| Prevalence of RA | 0.006 |
| Proportion Cases in Discovery | 0.422 |
| Proportion Cases in Test | 0.556 |
| Proportion of null SNPs | 0 |

**Table S6**: Parameters used for polygenic risk scoring power calculations

Polygenic scoring to investigate genetic overlap between RA and SCZ has power of 0.36, 0.90 and 0.99 to detect genetic effect correlations of magnitude 0.05, 0.10 and 0.15 respectively, at α = 0.05. This indicates our study is well powered to detect pleiotropic effects of the same magnitude observed in other pairs of disorders – for example Dudbridge estimates a genetic effect correlation between SCZ and bipolar disorder of 0.706 (95% CI: 0.513-0.897) assuming all SNPs have effects (Dudbridge, 2013).

Finally, we used software provided by Dudbridge to demonstrate that, assuming all SNPs have effects, we have 80% power here to detect a genetic effect correlation of 0.087. Assuming 1% of SNPs have effects, we have 80% power to detect a genetic effect correlation of 0.078. These are much less than genetic effect correlations estimated for canonically pleiotropic conditions with similar epidemiological relationships – such as the estimated genetic effect correlation between SCZ and bipolar disorder of 0.706 - and so it is likely that type II errors are not an issue in this analysis.

**Supplementary 7: Direction of effect for SNPs within the MHC**

A study using FDR-informed SNP ordering recently demonstrated that SCZ-associated SNPs acted in the same direction as those associated with Multiple Sclerosis more frequently than would be expected by chance – evidence of pleiotropy (Andreassen, Thompson, & Dale, 2014). Furthermore the authors demonstrated that this effect was driven by SNPs in the MHC region. We therefore repeated our direction of effect analysis above, focusing specifically on SNPs within the MHC (26 – 33mb on Chromosome 6).

After correcting for multiple testing, none of the results of sign tests for shared direction of effect were statistically significant (table S7). Therefore these results are consistent with those above, demonstrating a lack of genetic association between RA and SCZ, and nominal evidence for shared risk alleles.

| Clumped By | Threshold, p < | N SNPs | P, Pearson’s χ^2^ | Proportion SNPs in same direction |
| --- | --- | --- | --- | --- |
| RA | 0.01 | 95 | 0.201 | 0.526 |
|  | 0.1 | 132 | 0.025 | 0.515 |
|  | 0.2 | 145 | 0.090 | 0.531 |
|  | 0.3 | 149 | 0.129 | 0.523 |
|  | 0.4 | 152 | 0.152 | 0.520 |
|  | 0.5 | 152 | 0.152 | 0.520 |
| SCZ | 0.01 | 83 | 0.826 | 0.482 |
|  | 0.1 | 114 | 0.835 | 0.491 |
|  | 0.2 | 128 | 1.00 | 0.508 |
|  | 0.3 | 136 | 1.00 | 0.507 |
|  | 0.4 | 142 | 0.977 | 0.514 |
|  | 0.5 | 148 | 1.00 | 0.507 |

**Table S7**: Shared direction of effect between independent SCZ and RA risk alleles in the MHC region, across published GWAS,.

**Supplementary 8: Genetic Profile Risk Scoring**

SCZ genetic risk scores calculated using the panel of 542 SNPs proposed by Ayalew et al did not associate with RA status (Ayalew et al., 2012). We explored this further by investigating these SNPs within the most recent publically available RA GWAS (Stahl et al., 2010). Within an RA GWAS, the p-values for association with RA status were uniformly distributed (Kolmogorov-Smirnoff test p-value = 0.13). The SNP panel provided by Ayalew et al orders SNPs based on the genes they lie within. We therefore obtained this list of genes (n=42), and calculated gene-based p-values for rheumatoid arthritis using the summary data available from Stahl et al and the utility VEGAS (Liu et al., 2010). RA gene-based p-values were uniformly distributed across the 42 SCZ risk genes identified by Ayalew et al (Kolmogorov-Smirnoff test p-value = 0.604), indicating that genetic variation across these SCZ risk genes is not associated with RA status.

Displayed on a Manhattan plot of our RA case-control data after imputation and QC, no GPRS SNPs or their proxies show a suggestively significant association (α = 5x10^-5^) with RA (**supplementary figure 6**).

| 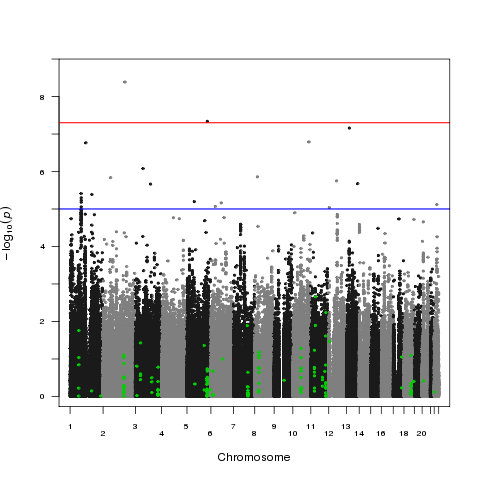 |
| --- |
| **Figure S6**: Manhattan plot of imputed SNPs in RA case-control analysis. GPRS542 SNPs and their proxies are highlighted in green. |

**References**

Andreassen, O. A., Thompson, W. K., & Dale, A. M. (2014). Boosting the power of schizophrenia genetics by leveraging new statistical tools. *Schizophr Bull, 40*(1), 13-17. doi: 10.1093/schbul/sbt168

Ayalew, M., Le-Niculescu, H., Levey, D. F., Jain, N., Changala, B., Patel, S. D., . . . Niculescu, A. B. (2012). Convergent functional genomics of schizophrenia: from comprehensive understanding to genetic risk prediction. *Mol Psychiatry, 17*(9), 887-905. doi: 10.1038/mp.2012.37

Dudbridge, F. (2013). Power and predictive accuracy of polygenic risk scores. *PLoS Genetics, 9*(3). doi: 10.1371/journal.pgen.1003348

Eyre, Steve, Bowes, John, Diogo, Dorothée, Lee, Annette, Barton, Anne, Martin, Paul, . . . Worthington, Jane. (2012). High-density genetic mapping identifies new susceptibility loci for rheumatoid arthritis. *Nature genetics, 44*(12), 1336-1340.

Lee, S. H., Yang, J., Goddard, M. E., Visscher, P. M., & Wray, N. R. (2012). Estimation of pleiotropy between complex diseases using single-nucleotide polymorphism-derived genomic relationships and restricted maximum likelihood. *Bioinformatics, 28*(19), 2540-2542. doi: 10.1093/bioinformatics/bts474

Liu, J. Z., McRae, A. F., Nyholt, D. R., Medland, S. E., Wray, N. R., Brown, K. M., . . . Macgregor, S. (2010). A versatile gene-based test for genome-wide association studies. *Am J Hum Genet, 87*(1), 139-145. doi: 10.1016/j.ajhg.2010.06.009

Stahl, Eli A., Raychaudhuri, Soumya, Remmers, Elaine F., Xie, Gang, Eyre, Stephen, Thomson, Brian P., . . . Plenge, Robert M. (2010). Genome-wide association study meta-analysis identifies seven new rheumatoid arthritis risk loci. *Nature genetics, 42*(6), 508-514. doi: 10.1038/ng.582

Wellcome Trust Case Control Consortium. (2007). Genome-wide association study of 14,000 cases of seven common diseases and 3,000 shared controls. *Nature, 447*(7145), 661-678. doi: 10.1038/nature05911
